# Incremental cost effectiveness of proton pump inhibitors for the prevention of non-steroidal anti-inflammatory drug ulcers: a pharmacoeconomic analysis linked to a case-control study

**DOI:** 10.1186/ar2577

**Published:** 2008-12-16

**Authors:** Harald E Vonkeman, Louise MA Braakman-Jansen, Rogier M Klok, Maarten J Postma, Jacobus RBJ Brouwers, Mart AFJ van de Laar

**Affiliations:** 1 Department of Rheumatology and Clinical Immunology, Medisch Spectrum Twente and University of Twente, Ariensplein 1, 7511 JX Enschede, The Netherlands; 2 Department of Psychology & Communication of Health & Risk, University of Twente, Citadel, 7500 AE Enschede, The Netherlands; 3 Groningen University Institute for Drug Exploration (GUIDE), Department of Social Pharmacy, Pharmacoepidemiology and Pharmacotherapy, Groningen University, Antonius Deusinglaan 1, 9713 AV Groningen, The Netherlands

## Abstract

**Introduction:**

We estimated the cost effectiveness of concomitant proton pump inhibitors (PPIs) in relation to the occurrence of non-steroidal anti-inflammatory drug (NSAID) ulcer complications.

**Methods:**

This study was linked to a nested case-control study. Patients with NSAID ulcer complications were compared with matched controls. Only direct medical costs were reported. For the calculation of the incremental cost effectiveness ratio we extrapolated the data to 1,000 patients using concomitant PPIs and 1,000 patients not using PPIs for 1 year. Sensitivity analysis was performed by 'worst case' and 'best case' scenarios in which the 95% confidence interval (CI) of the odds ratio (OR) and the 95% CI of the cost estimate of a NSAID ulcer complication were varied. Costs of PPIs was varied separately.

**Results:**

In all, 104 incident cases and 284 matched controls were identified from a cohort of 51,903 NSAID users with 10,402 NSAID exposition years. Use of PPIs was associated with an adjusted OR of 0.33 (95% CI 0.17 to 0.67; p = 0.002) for NSAID ulcer complications. In the extrapolation the estimated number of NSAID ulcer complications was 13.8 for non-PPI users and 3.6 for PPI users. The incremental total costs were € 50,094 higher for concomitant PPIs use. The incremental cost effectiveness ratio was € 4,907 per NSAID ulcer complication prevented when using the least costly PPIs.

**Conclusions:**

Concomitant use of PPIs for the prevention of NSAID ulcer complications costs € 4,907 per NSAID ulcer complication prevented when using the least costly PPIs. The price of PPIs highly influenced the robustness of the results.

## Introduction

Treatment with non-steroidal anti-inflammatory drugs (NSAIDs) is known to be complicated by serious gastrointestinal toxicity. NSAIDs impair prostaglandin-dependent gastric mucosal protective mechanisms. When these defences have been breached, a second wave of injury caused by luminal gastric acid may facilitate deep ulceration, eventually causing ulcer bleeding and perforation [1]. Several strategies have been developed to prevent NSAID ulcers [2,3]. In clinical trials different selective cyclooxygenase (COX)-2 inhibitors, proton pump inhibitors (PPIs), high dose histamine-2 receptor antagonists and prostaglandin analogues have been shown to decrease the risk for NSAID ulcers. However, few strategies have been directly compared, and for most a formal cost effectiveness analysis is lacking.

In a previous study, we found that concomitant use of PPIs was associated with a significant reduction of serious NSAID ulcer complications [4]. In a further study, we calculated the direct medical costs of hospitalisation for serious NSAID ulcer complications [5]. The objective of the present study was to extend these analyses by performing a pharmacoeconomical evaluation [6]. Such an assessment is relevant to furnish clinical guidelines (for example, on standard concomitant PPI use with NSAIDs) with the appropriate pharmacoeconomic information.

## Materials and methods

The pharmacoeconomic evaluation was linked to a 26-month observational study conducted in the Enschede healthcare district of The Netherlands, in which a cohort of 51,903 NSAID users is served by 14 pharmacies and a single large teaching hospital, equipped with all diagnostic and therapeutic facilities [4]. All drug prescriptions for the population are registered via electronic prescription records. The majority of drugs, including NSAIDs, are provided by the patients' own pharmacy, with direct reimbursment by the state healthcare system. The cohort of NSAID users can therefore continuously be identified using the electronic prescription records.

The study used a nested case-control design. From November 2001 until December 2003, we identified all NSAID users with serious NSAID ulcer complications. Serious NSAID ulcer complications were defined as ulcerations of the stomach or proximal duodenum causing perforation, obstruction or bleeding during the use of NSAIDs, necessitating hospitalisation of the patient. Patients were identified by endoscopy or abdominal surgery and were included in the study if they used NSAIDs at the time a gastroduodenal ulcer was diagnosed. For each serious NSAID ulcer complication, the patient was invited to complete a questionnaire on his/her sociodemographic characteristics, actual and recent medication, comorbidity and medical history. When applicable for reasons of verification of the questionnaires, we reviewed medical charts, as well as endoscopy, surgery and pathology reports. Medication use prior to and during hospitalisation as reported by the patient, was verified by reviewing prescription records provided by the in-hospital and community based pharmacies.

Controls were retrieved from the remaining cohort of NSAID users who had not developed serious NSAID ulcer complications at the time of ulcer occurrence in each of the cases. For selecting controls, index dates were defined as the day on which a NSAID ulcer complication was diagnosed in each of the cases. Controls were frequency matched by sex and age, and had to be using an NSAID on the index date. Selected controls were invited to complete the same questionnaire. Medication use as reported by the controls was verified by reviewing prescription records. The study was approved by the Institutional Ethical Review Board. All patients gave informed consent.

Omeprazole ≥ 20 mg, pantoprazole ≥ 20 mg, lansoprazole ≥ 15 mg, esomeprazole ≥ 20 mg and rabeprazole ≥ 20 mg were considered PPIs in adequate dosage for the prevention of NSAID ulcers.

### Outcome

Because a patient could theoretically have more than one episode with serious NSAID ulcer complications, the preferred unit of analysis was the episode with a serious NSAID ulcer complication rather than the patient. The outcome of interest was the occurrence of a serious ulcer complication during NSAID use.

### Costs

The measure of interest was the cost of PPI treatment and the cost(s) of medical treatment of serious NSAID ulcer complications. Included in the costs of medical treatment were all direct medical costs made during hospitalisation [5]. No information was available for costs of general practitioner visits, outpatient treatments by medical specialists or drug therapy. The costs for NSAID therapy and costs related to that therapy were not taken into account as these costs are expected to be similar in both treatment groups. Non-medical costs (for example, those related to work absenteeism) were not included.

Hospital service utilisation was determined using standard hospital administrative records and included the number of intensive care and standard care in-patient days, emergency department care, ambulance transportation, transfusion of blood products, endoscopies, surgery, (radio)diagnostic procedures, and laboratory tests. Table 1 lists all direct medical costs that were included in the analysis, presenting the method of valuation, the cost price per unit and its source. Unit costs were derived from the Dutch manual for costing [7], the Dutch tariff book for medical specialists [8], the Dutch tariff list for hospitals [9], and Dutch list prices for the various PPIs [10]. All prices are in € (Euros) at 2003 values. Unit costs for blood products were derived from the 2003 standard cost prices of blood products as determined by the Sanquin Blood Supply Foundation in The Netherlands [7]. To calculate direct medical costs, health resource use was multiplied by unit-cost estimates.

**Table 1:** Categories, methods and sources for valuation of unit costs [7-10]

**Categories**	**Unit of resource**	**Source of estimate**	**Unit cost (€)**
PPI (defined daily dose):			
Omeprazole, generic: 20 mg	Monthly costs	Pharmacotherapeutic Compass 2007	11.30
Lansoprazole (Prezal^®^): 30 mg	Monthly costs	Pharmacotherapeutic Compass 2007	29.71
Omeprazole (Losec^®^): 20 mg	Monthly costs	Pharmacotherapeutic Compass 2007	29.85
Rabeprazole (Pariet^®^): 20 mg	Monthly costs	Pharmacotherapeutic Compass 2007	31.75
Pantaprazole (Pantozol^®^): 40 mg	Monthly costs	Pharmacotherapeutic Compass 2007	36.41
Esomeprazole (Nexium^®^):30 mg	Monthly costs	Pharmacotherapeutic Compass 2007	39.37
Hospital admission:			
standard care	Number of days	Cost manual of Oostenbrink	337.00
Intensive care	Number of days	Cost manual of Oostenbrink	1,684.00
Emergency department	Number of visits	Cost manual of Oostenbrink	139.00
Ambulance transportation:			
Emergency	Number of transports	Cost manual of Oostenbrink	443.00
Regular	Number of transports	Cost manual of Oostenbrink	212.00
Blood products:			
Packed cells	Number of units	Cost manual of Oostenbrink	179.00
Blood platelets	Number of units	Cost manual of Oostenbrink	81.00
Fresh frozen plasma	Number of units	Cost manual of Oostenbrink	154.00
Endoscopy	Number of procedures	Tariff list hospitals	369.71
Surgery:			
Suture of perforation	Number of operations	Tariff list hospitals	870.79
Partial stomach resection	Number of operations	Tariff list hospitals	1,945.15
Total stomach resection	Number of operations	Tariff list hospitals	3,414.66
Cholecystectomy	Number of operations	Tariff list hospitals	944.55
Abdominal abscess drainage	Number of operations	Tariff list hospitals	662.00
(Radio)diagnostic procedures:			
Plain X-ray	Number of procedures	Tariff list hospitals	134.01
CT scan: abdomen	Number of procedures	Tariff list hospitals	198.85
CT scan: thorax	Number of procedures	Tariff list hospitals	228.85
MRI	Number of procedures	Tariff list hospitals	228.85
Abdominal ultrasound	Number of procedures	Tariff list hospitals	70.30
Cardiac ultrasound	Number of procedures	Tariff list hospitals	73.01
Vascular ultrasound	Number of procedures	Tariff list hospitals	58.54
Radionucleotide: total skeleton	Number of procedures	Tariff list hospitals	151.74
Radionucleotide: embolism	Number of procedures	Tariff list hospitals	359.58
Radionucleotide: abscess	Number of procedures	Tariff list hospitals	651.84
Pulmonary function test	Number of procedures	Tariff list hospitals	61.40
Electrocardiogram	Number of procedures	Tariff list hospitals	34.23
Laboratory tests:			
Standard set of laboratory tests	Number of procedures	Tariff list hospitals	13.85
Microbiology culture	Number of procedures	Tariff list hospitals	30.97
Pathology testing	Number of procedures	Tariff list hospitals	49.33

CT, computed tomography; MRI, magnetic resonance imaging.

### Statistics

In our previous study, multivariate analysis using logistic regression was performed on the occurrence of serious ulcer complications in patients using NSAIDs [4]. The adjusted odds ratio (OR) was calculated for serious NSAID user complications with concomitant PPIs compared with serious NSAID user complications without PPIs. The estimated OR for the occurrence of a serious NSAID ulcer complication with concomitant PPIs compared with no PPIs can be interpreted as approaching the corresponding relative risk (RR). Exposure times did not differ significantly between cases and controls (median 1.13 months). As the OR is assumed to correspond with the RR, the number of serious NSAID ulcer complications possibly prevented by the use of PPIs can be approximated by using: ((1-1/OR) × observed cases). Subsequently, we inserted this assumption into the pharmacoeconomic analysis to estimate the proportion of serious ulcer complications in NSAID users that might have been averted by adding a PPI.

The mean total direct costs per occurrence of a serious NSAID ulcer complication were calculated and 95% confidence intervals (95% CIs) were estimated using a bootstrap procedure [5].

Statistical analyses were performed with SPSS for Windows, version 12.0.1 (SPSS, Chicago, IL, USA). Bootstrap analyses were performed using the software package S-plus (TIBCO Software Inc., Palo Alto, California, USA) professional version 6.0.

### Cost effectiveness

To calculate the incremental cost effectiveness ratio (expressed as net costs per serious NSAID ulcer complication prevented) we extrapolated the data (by multiplication) to 1,000 patients using concomitant PPIs and 1,000 patients not using PPIs for the duration of 1 year. For effectiveness we used serious NSAID ulcer complications as the main outcome measure. The number of cases was calculated using the risk estimates of the first part of this study. Costs were calculated by multiplying the number of serious NSAID ulcer complications with the cost of a serious NSAID ulcer complication in combination with the costs of PPI treatment. The incremental cost effectiveness ratio was calculated by the difference in total direct medical costs divided by the difference in number of serious NSAID ulcer complication for the group using concomitant PPIs and the group not using concomitant PPIs.

To test the robustness of the results, two approaches were used. The first one takes the uncertainty of the estimates of risk for serious NSAID ulcer complications into account (95% CI of the OR) as well as the uncertainty for the estimate of the cost of a serious NSAID ulcer complication (95% CI of the cost estimate). To show this uncertainty we used the extreme estimates for both the most positive and the most negative options for concomitant PPI therapy and NSAID use. The second approach was used to show the impact of varying the cost of PPI treatment on the expected incremental cost effectiveness ratio.

## Results

During the 26-month study period 104 incident cases with serious NSAID ulcer complications were observed in a cohort of 51,903 NSAID users with a cumulative 10,402 patient years of NSAID use (Table 2) [5]. There were no cases with more than one event during the observational period. Data for these cases was retrieved from questionnaires and hospital administrative records. The typical case is an older patient, mean age at diagnosis 70.4 years (SD 16.7; youngest 22 years, eldest 98 years), 55.8% were female. In 86 (82.7%) patients the clinical presentation was that of an acute upper gastrointestinal bleeding or perforation. In 53 (51%) patients the ulcer was located in the stomach, 34 (32.7%) had a duodenal ulcer and 11 (10.6%) had both gastric and duodenal ulcers. The ulcer perforated in 14 (13.5%) patients. Mortality due to serious NSAID ulcer complications was high; 11 (10.6%) patients died in hospital, and another 4 (3.8%) died within 3 months of the diagnosis. The median duration of hospitalisation was 9.0 days (range 1 to 87 days); 11 patients spent up to 7 days in the intensive care unit and 1 patient spent 26 days. Most patients (88; 84.8%) underwent at least 1 diagnostic endoscopy. A surgical procedure was performed in 18 (17.3%) patients. The estimated mean total direct cost of a serious NSAID ulcer complication was € 8,375 per patient (95% CI 7,067 to 10,393) [5].

**Table 2:** Demographic characteristics, medical history and current medication for cases and controls

	**Cases** **(n = 104)**	**Controls** **(n = 284)**	**OR**	**95% CI**	**p Value**
Demographic characteristics:					
Age at diagnosis (years)	70.4 (16.7)	67.1 (14.3)	-	-	0.06
Sex (female)	58 (55.8%)	163 (57.4%)	0.95	0.60 to 1.47	0.78
Smoking	28 (26.9%)	51 (18%)	1.96	1.15 to 3.37	0.01
Alcohol (glasses/week)	9.6 (33.2)	6.2 (8.6)	-	-	0.12
Medical history:					
Heart failure	26 (25.0%)	32 (11.3%)	2.63	1.48 to 4.67	0.001
Renal insufficiency	16 (15.4%)	15 (5.3%)	3.26	1.55 to 6.86	0.001
Myocardial infarction	20 (19.2%)	32 (11.3%)	1.88	1.02 to 3.45	0.04
Stroke	18 (17.3%)	28 (9.9%)	1.91	1.01 to 3.63	0.04
Diabetes mellitus	16 (15.4%)	33 (11.6%)	1.38	0.73 to 2.64	0.32
Previous gastrointestinal ulcers	16 (15.4%)	33 (11.7%)	1.37	0.72 to 2.60	0.34
Rheumatoid disease, including OA	42 (40.4%)	97 (34.2%)	1.31	0.82 to 2.07	0.26
Medication:					
Non-selective NSAIDs	86 (82.7%)	222 (78.2%)	1.33	0.75 to 2.39	0.33
Selective COX-2 inhibitors	17 (16.3%)	50 (17.6%)	0.91	0.50 to 1.67	0.77
Preferential COX-2 inhibitors	1 (1.0%)	12 (4.2%)	0.22	0.03 to 1.71	0.20
Proton pump inhibitors	14 (13.5%)	77 (27.1%)	0.42	0.23 to 0.78	0.005
H2RAs	4 (3.8%)	9 (3.2%)	1.22	0.37 to 4.06	0.74
Misoprostol	8 (7.7%)	20 (7.0%)	1.10	0.47 to 2.58	0.83
Low dose aspirin (≤ 100 mg/day)	32 (30.8%)	69 (24.3%)	1.39	0.84 to 2.28	0.20
Coumarin	14 (13.5%)	19 (6.7%)	2.17	1.05 to 4.51	0.04
SSRIs	6 (5.8%)	9 (3.2%)	1.87	0.65 to 5.39	0.24
Corticosteroids	14 (13.5%)	32 (11.3%)	1.23	0.63 to 2.40	0.55

Scores are mean values (standard deviation) or number of patients (%). CI, confidence interval; COPD, chronic obstructive pulmonary disease; COX, cyclooxygenase; H2RA, histamine receptor-2 antagonist; NSAID, non-steroidal anti-inflammatory drug; OA, osteoarthritis; OR, unadjusted odds ratio; SSRI, selective serotonin re-uptake inhibitor.

From the remaining cohort of NSAID users a total of 284 controls were retrieved, frequency matched by age and sex, who were using NSAIDs on the index date. Demographic characteristics, comorbidities and current medication use are summarised in Table 2. Mean age was slightly lower in the controls than in the cases because insufficient numbers of controls could be found for some of the more senior patients.

Concomitant use of PPIs was significantly higher in the controls than in the cases (cases 14 (13.5%) and controls 77 (27.1%); p = 0.005). Use of selective COX-2 inhibitors was comparable (cases 17 (16.4%) and controls 50 (17.6%); p = 0.77). Use of the preferential COX-2 inhibitor meloxicam differed, but not significantly, and numbers were small (cases 1 (1%) and controls 12 (4.2%); p = 0.20). The adjusted OR for serious NSAID ulcer complications was 0.33 (95% CI 0.17 to 0.67; p = 0.002) for concomitant use of PPIs compared with no PPIs [4]. Both groups differed in their risk for developing NSAID ulcer complications. The group using concomitant PPIs significantly more often used chronic NSAID therapy (more than 3 months continuously), concomitant steroids, had a medical history of anaemia, and of previous gastroduodenal events.

In the extrapolation to 1,000 patients not using concomitant PPIs, the estimated number of serious NSAID ulcer complications was 13.8 (95% CI 13.7 to 13.8). In the extrapolation to 1,000 patients using concomitant PPIs, the estimated number of serious NSAID ulcer complications was 3.6 (95% CI 3.56 to 3.64). Costs were calculated by multiplying the number of serious NSAID ulcer complications with the cost of a serious NSAID ulcer complication (€ 8,375) in combination with the costs of the cheapest PPI treatment (generic omeprazole, estimated at € 135,600 (1,000 × € 11.30 × 12 months)). Therefore the total costs associated with serious NSAID ulcer complications was (13.8 × € 8,375) = € 115,676 (95% CI 114,874 to 116,493) for the group not using concomitant PPIs and ((3.6 × € 8,375) + € 135,600) = € 165,770 (95% CI € 160,789 to € 173,444) for the group using concomitant PPIs (Table 3). The incremental cost effectiveness ratio after 1 year of follow-up was (€ 50,094/10.2) = € 4,907 per serious NSAID ulcer complication prevented, when using the least costly PPI.

**Table 3:** Comparison of the number of serious NSAID ulcer complications and associated costs in the two extrapolations (all using PPIs vs none using PPIs)

	**No PPI users** **(n = 1,000)**	**PPI users** **(n = 1,000)**	**Difference**
No. of complications (95% CI)	13.8 (13.7 to 13.9)	3.60 (3.56 to 3.64)	10.2
Costs^a ^(95% CI)	€ 115,676 (114,874 to 116,493)	€ 165,770 (160,789 to 173,444)	€ 50,094

^a^Cost of cheapest concomitant PPI (generic omeprazole) was taken into account. NSAID, non-steroidal anti-inflammatory drug; PPI, proton pump inhibitor.

In Table 4, the cost effectiveness ratio is shown with different monthly costs for the concomitant PPI used. It can be seen that the estimated upper (6,290) and lower (2,813) limit for the incremental cost effectiveness ratio does not differ much from the point estimate, indicating that with the current estimate of the risk of serious NSAID ulcer complications and the estimate of costs associated with those serious NSAID ulcer complications, no large differences in incremental cost effectiveness should be expected. However, changing the monthly costs of PPI-treatment itself does markedly increase the incremental cost effectiveness ratio, as is shown in Table 4. When using the most expensive option (on a 2007 defined daily dose (DDD) level), esomeprazole (Nexium^®^), the incremental cost effectiveness ratio is € 37,899 per serious gastrointestinal event prevented.

**Table 4:** Expected monthly costs (based on defined daily dose) and cost effectiveness for different PPIs at 2007 price levels [10]

**Drug**	**Defined daily dose^a^**	**Monthly costs (November 2006)**	**Cost effectiveness ratio (lower and upper limit)^b^**
Generic omeprazole	20 mg	€ 11.30	4,907 (2,813 to 6,290)
Lansoprazole (Prezal^®^)	30 mg	€ 29.71	26,545 (24,327 to 28,051)
Omeprazole (Losec^®^)	20 mg	€ 29.85	26,709 (24,491 to 28,217)
Rabeprazole (Pariet^®^)	20 mg	€ 31.75	28,943 (26,711 to 30,463)
Pantaprazole (Pantozol^®^)	40 mg	€ 36.41	34,420 (32,157 to 35,971)
Esomeprazole (Nexium^®^)	30 mg	€ 39.37	37,899 (35,617 to 39,470)

^a^The daily dosing schedule on which the cost effectiveness ratio is based, may not always reflect the actual dosages prescribed in clinical practice; ^b^cost effectiveness is expressed as costs (€) per serious NSAID ulcer complication prevented: lower and upper limit are the results of the sensitivity analyses.

NSAID, non-steroidal anti-inflammatory drug.

## Discussion

In this analysis we found that in NSAID users, concomitant use of PPIs costs € 4,907 per serious NSAID ulcer complication prevented when using the least costly PPI. This pharmacoeconomic analysis extends the findings of our previous clinical study in NSAID users, in which concomitant use of PPIs was associated with a lower incidence of serious NSAID ulcer complications compared with not using PPIs [4].

The incremental cost analysis was performed from a health care perspective and only direct medical costs made during hospitalisation were available. Inclusion of extramural direct medical costs (for example, general practitioner visits and outpatient treatments), direct non-medical costs (for example, travel to and from the hospital) and indirect non-medical costs (for example, those related to work absenteeism) might possibly strengthen the favourable economic profile of concomitant PPI use in NSAID users, compared with not using concomitant PPIs.

For estimation of the effects of using concomitant PPIs, we extrapolated case-control data from a cohort of NSAID users on the occurrence of serious NSAID ulcer complications in patients using concomitant PPIs and in patients not using PPIs. Based on obtained history, the group using concomitant PPIs would be expected to have a higher risk for developing NSAID ulcer complications than the group without PPIs. Therefore the effect size of concomitant use of PPIs may have been underestimated, which would further strengthen the favourable economic profile of concomitant PPI use.

Using the OR as an approximation of the RR may overestimate the favourable economic profile of concomitant PPI use in NSAID users, if the risk of serious NSAID ulcer complications is not very low in the population studied [11]. In the present study the risk of overestimation is negligible as the incidence rate of serious NSAID ulcer complications was approximately 1% per year of NSAID use, which is in concurrence with the current literature [12,13].

In this analysis, we found that an increase in PPI costs markedly increases the incremental cost effectiveness ratio. Cost effectiveness of concomitant use of PPIs in NSAID users may be less favourable if NSAID users switch to more expensive brand name drugs instead of using generic preparations. Due to active legislation it is probable, however, that the majority of patients will use the cheapest treatment option of generic omeprazole. The incremental cost effectiveness ratio of concomitant use of PPIs in NSAID users may be raised further by inappropriate use of PPIs (for example, on demand use during continued NSAID use), or in combination with other gastroprotective strategies (for example, high dose histamine receptor-2 antagonists or misoprostol). Furthermore, PPI use is sometimes continued indefinitely after its necessity has ended, such as after NSAID treatment has stopped.

In the present study, concomitant PPIs were found to cost € 4,907 per averted serious NSAID ulcer complication in NSAID users with one or more risk factors for NSAID gastrointestinal toxicity. According to Spiegel *et al*. [14], generic non-selective NSAIDs alone were optimally cost effective for patients at low risk for NSAID-related gastrointestinal complications, while in patients with one or more risk factors adding a PPI to a non-selective NSAID was the dominant strategy. In contrast, another study found selective COX-2 inhibitors to be most cost effective, while a third study found both strategies to be cost effective, dependent on the baseline risk [15,16]. In a comprehensive systematic review with economic modelling, both H_2 _receptor antagonists and PPIs were found to be cost effective for avoiding endoscopic ulcers in patients requiring long-term NSAID therapy. Furthermore, prescribing H_2 _receptor antagonists was found to be possibly cost effective in all patients requiring NSAIDs [17,18]. While these findings from previous studies vary, they all used actual primary clinical data from trials and applied them to an economic model. These data may however not always be generalised outside the controlled environment of the clinical trials. In the present study, we therefore prospectively observed a large cohort of real NSAID users, calculated the actual direct medical costs made by patients with serious NSAID ulcer complications, and conducted a subsequent nested case-control study to evaluate the different gastroprotective strategies used [4,5]. Although observational studies are subject to possible bias, linking pharmacoeconomical analyses to case-control studies may be a valuable addition to the ongoing discussion on cost effectiveness of preventive pharmacotherapy.

## Conclusion

In this pharmacoeconomical analysis of NSAID users, concomitant use of PPIs costs € 4,907 to prevent one serious NSAID ulcer complication if generic omeprazole is used. However, using a more expensive PPI will increase the cost of preventing one serious NSAID ulcer complication to more than € 25,000.

## Abbreviations

COX-2: cyclooxygenase-2; NSAIDs: non-steroidal anti-inflammatory drugs; PPIs: proton pump inhibitors.

## Competing interests

The authors declare that they have no competing interests.

## Authors' contributions

All authors contributed significantly to the writing of the paper. MJP, JRBJB and MvdL conceived the study, and participated in its design and coordination. HEV and MvdL conducted the case-control study. HEV, RMK, MJP, JRBJB and MvdL conducted the cost of illness study. HEV, LMB-J, RMK and MJP conducted the pharmacoeconomical analysis. All authors read and approved the final manuscript.
